# Ginkgo Biloba Extract Reduces Cardiac and Brain Inflammation in Rats Fed a HFD and Exposed to Chronic Mental Stress through NF-*κ*B Inhibition

**DOI:** 10.1155/2022/2408598

**Published:** 2022-05-29

**Authors:** Lijun Zhang, Guo Li, Shuhui Tao, Pengyan Xia, Naveed Chaudhry, Shawn Kaura, Sara Saymuah Stone, Meiyan Liu

**Affiliations:** ^1^Department of Psycho-Cardiology, Beijing Anzhen Hospital Affiliated to Capital Medical University, Beijing 100029, China; ^2^Henan Medical School, Henan University, Kaifeng 475001, China; ^3^Institute for Stem Cell and Regenerative, Chinese Academy of Sciences, Beijing, China; ^4^Lake Erie College at Osteopathic Medicine at Seton Hill, 20 Seton Hill Drive, Greensburg, PA 15601, USA; ^5^Department of Neurosurgery, Wayne State University School of Medicine, Detroit, MI, USA

## Abstract

**Background:**

Cardiac and brain inflammation can lead to a host of deleterious health effects. Our formal experimental research showed that *Ginkgo Biloba Extract* (GBE) contributed to the reduction of inflammation in mice with myocardial infarction along with depression. This study is aimed at expanding on these findings via analysis of the cardiac and brain inflammation, which was prevented by GBE in rats suffering with a high-fat diet (HFD) combined with unpredictable chronic mild stress (UCMS).

**Methods:**

Fifty male Wistar rats were randomly divided into 5 groups treated with normal diet, UCMS, HFD, HFD+UCMS, or HFD+UCMS+GBE respectively. Rats treated with HFD were fed a high-fat diet for 10 or 13 weeks. Rats treated with UCMS were exposed to 8 types of chronic physical and psychological stressors for 10 or 13 weeks. The HFD+UCMS+GBE group was given GBE via intragastric gavage for 8 consecutive weeks. Sucrose preference was established for the assessment of depressive behaviors. The heart function was evaluated by echocardiography. The rats were terminated at the end of the 10^th^ or 13^th^ week. The blood was used for detecting low-density lipoprotein cholesterol (LDL-c) and total cholesterol (TCHO) by the kit instructions; Helper T Lymphocytes (TH cells, CD3^+^CD4^+^) by flow cytometry; and Interleukin- (IL-) 1*β*, IL-37, IL-38, NT-proBNP, hs-cTNI, and Ischemia-modified albumin (IMA) by enzyme-linked immunosorbent assay (ELISA). The cardiac tissues were used for detecting IL-1*β*, nuclear factor kappa B (NF-*κ*B), inhibitor molecule protein (I*κ*B), and IL-1 receptor (IL-1R) by ELISA and P65, P-P65, I*κ*B, and phosphorylated inhibitor molecule protein *α* (P-I*κ*B*α*) for western blotting. Cortex tissues were used for detecting 8-iso-prostaglandinF2*α* (8-iso-PGF2*α*) by ELISA. Oil Red staining was carried out to evaluate the lipid deposits in the rats' aortic arteries. Sirius Red staining was performed to display collagen fibers in the arteries. Hematoxylin and Eosin (HE) staining was applied to reveal pathological changes to arteries and cardiac tissue. Immunohistochemical staining was employed to assess the distribution of inflammatory cytokine IL-1*β* in arteries and cardiac tissues. Transmission Electron Microscopy (TEM) was performed to observe the ultrastructure of hippocampal cornu ammonis (CA)1 (CA1) neurons.

**Results:**

In the rats with HFD+UCMS+GBE, over 13 weeks, GBE exerted a protective role of both the heart and brain, by attenuating cardiac inflammation and brain oxidative stress. Levels of Helper T lymphocytes and serum anti-inflammatory cytokines involving IL-37 and IL-38 were all elevated, and the depressive behaviors of HFD+UCMS rats were attenuated by GBE. This protective role was accomplished via inhibition of the canonical NF-*κ*B signaling pathway, through downregulation of the expressions of P-P65 and P-I*κ*B-*α* in the heart, hippocampus, cortex, and hypothalamus.

**Conclusions:**

This study suggests that GBE poses a protective role from the various pathologies associated with high-fat diets, unpredictable chronic mild stress, and depression, possibly via improving peripheral immunity and reducing cardiac and brain inflammation.

## 1. Introduction

Coronary artery disease (CAD) is a critical threat to human health, and tens of millions of people dying of acute coronary artery syndrome and acute myocardial infarction every year globally [1, 2]. To a certain extent, atherosclerosis, caused by high-fat diet, plays a vital role in inducing and worsening CAD. Therefore, CAD is closely associated with consumption of a high-fat diet. A high-fat diet induces lipid metabolism dysfunction by increasing the concentration of total cholesterol (TCHO) and low-density lipoprotein cholesterol (LDL-c), being oxidized to ox-LDL-c which is swallowed by macrophage in the vessels. Consequently, a widely validated body of macrophage turns into foam cells, and white blood cells notably monocytes and lymphocytes migrate to the intermedia of the vessel, releasing inflammatory factors into the blood stream, namely, Interleukin- (IL-) 1*β*, IL-4, IL-6, and IL-10. The inflammatory factors flow in the circulation system triggering the activation of the immunity system, blood coagulation system, neuroendocrine system, and even the hypothalamus-pituitary-adrenal (HPA) axis. Gradually, amount of plaque generated in the vessel, resulting in the atherosclerosis which is the initial factor of coronary artery disease. What is worse, when high-fat diet coexists with chronic stress, the cardiac function and psychological status might be worsen via the immune system imbalance. Therefore, high-fat diets are associated with both cardiac and brain injuries [3, 4].

Moreover, chronic mental stress, which has been found to be nearly unavoidable in the modern era, has an equally unfavorable influence on heart and brain functions [5], leading to or worsening preexisting CAD or depression directly. Numerous clinical studies have been reported that have significant comorbidity of coronary artery disease as well as psychological disease, namely, depression and/or anxiety [6], and this concept has been more widely accepted. Therefore, it is necessary to further explore the pathophysiological mechanisms of the interactions between the heart and brain. However, the interactions are quite complex, involving immune imbalance, inflammation, the serotonin system, HPA axis hyperactivity, sympathetic and parasympathetic nerve imbalance, metabolic dysregulation, microcirculation dysfunction, platelet aggregation/thrombosis, mitochondrial dysfunction, etc. [7].

ox-LDL could be reduced by pretreatment of Ginkgo Biloba Extract (GBE) via increasing the amount of nitric oxide, thus protecting the endothelial function and defending from atherosclerosis [8]. In addition, GBE could relieve atherosclerosis by decreasing plasma lipid via reducing the expression of connexin 43 protein [9]. In our previous research, we have demonstrated the promising effect of GBE and Ginkgo B in attenuating depressive behaviors in mice with myocardial infarction (MI) through inflammation suppression [10].

Thereafter, we carried out the present study aimed at establishing the therapeutic effects of GBE on heart and brain inflammation in rats, which were fed with a high-fat diet (HFD) and exposed to unpredictable chronic mild stress (UCMS). In the research progress, firstly, we established rat models with HFD and/or UCMS, and some rats were treated with GBE. Secondly, we evaluated the rats' heart function by echocardiography and depressive behaviors by a source preference test. Thirdly, we detected the inflammatory response of rats by enzyme-linked immunosorbent assay (ELISA), western blotting, flow cytometry, Hematoxylin and Eosin (HE) staining, Immunohistochemical staining, and Transmission Electron Microscopy (TEM).

## 2. Materials and Methods

### 2.1. Subjects

Fifty male Wistar rats (Charles River Laboratories, No.1100112011050133), aged 8 weeks and weighing 240~260 g, were housed under a 12-hour light-dark cycle, with food and water *ad libitum* (room temperature, 19-22°C; humidity, 50-60%). After adaptation for one week, the rats were divided into 5 groups: Control group, Control+UCMS group, HFD group, HFD+UCMS group, and HFD+UCMS+GBE group (*n* = 10 in each group). The rats in the HFD, HFD+UCMS, and HFD+UCMS+GBE groups were fed a high-fat diet (81.3% basic feed, 10% lard, 5% sucrose, 3% cholesterol, 0.5% sodium cholate, and 0.2% propylthiouracil; Beijing Ke'aoXieli Feed Co., Ltd., No. 2020090802), while those in the Control group had a regular diet (Beijing HFK Bioscience CO. LTD, Beijing, China) for 10- or 13-week treatments. According to the experience of using this kind of high-fat fodder, the rats would exhibit obvious lipid deposits by Oil Red staining and collagen fibers during 10-13 weeks of feeding, therefore, we chose to end the experiment at the 10^th^ week and 13^th^ week. A vitamin D injection was given to all rats receiving HFD over the first three weeks, with doses of 700,000 U/kg once a week.

All the rats underwent a weekly weighing, behavioral tests, and echocardiography before termination. Half of the rats in each group were terminated after 10 weeks of the study, with the remainder terminated after 13 weeks. The rats were anesthetized by injecting 3% pentobarbital sodium and then terminated the morning after a 10-hour fast. Blood samples, the aorta, the heart, and the brain were collected for the procedures described below.

All the animals in this experiment were cared for according to the animal care guidelines formulated by the Institute for Laboratory Animal Research of Capital Medical University affiliated Beijing Anzhen Hospital. The experiment was approved by the Animal Ethical and Welfare Committee of Capital Medical University affiliated Beijing Anzhen Hospital (No. 2019036X).

### 2.2. GBE Treatments

Rats in the HFD+UCMS+GBE group were given GBE by intragastric gavage (GBE was from Ginkgo biloba dripping pills, provided by WanBangDe Pharmaceutical Group Co., Ltd., Lot No. A01J200304, Zhejiang, China) and 40 mg/kg/day of GBE [11, 12] for 8 consecutive weeks. Other rats were given 1 ml 0.9% saline by intragastric gavage. The preparation methods, properties, accurate quantitative determination method, indications or functions, usage, and dosage of Ginkgo biloba dripping pills were all introduced in the Chinese Pharmacopoeia [11].

### 2.3. Chronic and Mild Stress

Eight types of unpredictable chronic mild stress conditions were applied to the rats in the Control+UCMS, HFD+UCMS, and HFD+UCMS+GBE groups, involving flashlight stress for 30 minutes (min), sound stress for 30 min, water stress for 30 min, 4°C cold stress for 10 min, single cage stress for 24 hours, 45° incline for 30 min, tie stress for 30 min, and crowding stress for 30 min. A single condition of stress was presented each day of the week, respectively, for either 10 weeks or 13 weeks. Moreover, the same stress condition was not repeated within the same week to avoid adaptation of the rats.

### 2.4. Sucrose Preference Test

A sucrose preference test was performed to measure depressive behaviors in rats at the end of the 10^th^ and 13^th^weeks, as described in our previous published study [13]. The rat was kept alone in a cage for 24 hours while going through the sucrose preference test. Two bottles were placed on opposite sides of each cage, respectively, one bottle of pure water on the left and the other with 1% sucrose water on the right; the positions of bottles were exchanged after 12 hours. After 24 hours for adaptation, the test was performed for a further 24 hours, before recording the consumption of pure water, sucrose water, and total water. The sucrose preference was calculated by (sucrose consumption/total water consumption) × 100%.

### 2.5. Echocardiography

At the end of the 10^th^ and 13^th^ weeks, echocardiography was performed using a Visualsonics Vevo 2100 (FUJIFILM VisualSonics Inc., Toronto, Canada) with a 20 MHz linear transducer, to evaluate cardiac systolic and diastolic function in all rat groups. M-mode ultrasound was used for measuring the diameter of the aorta root and aorta ascendens, left ventricular ejection fraction (LVEF), left ventricular fractional shortening (LVFS), left ventricular (LV) mass correction, maximum flow velocity of peak E wave of Mitral valve (MV E), maximum flow velocity of peak A wave of Mitral valve (MV A), and the MV E/A ratio. During echocardiography, the rats were anesthetized with 2% isoflurane (1 l/min O_2_ gas).

### 2.6. Flow Cytometry

At the end of the 13^th^ week, the fresh blood of rats in all the five groups was collected for flow cytometry. Flow cytometry was conducted using a Becton Dickinson (BD): LSRFortessa (Becton, Dickinson and Company, New Jersey, USA), to determine the levels of immune cells including Helper T Lymphocytes (T_H_ cells, CD3^+^CD4^+^). The antibodies included anti-Rat-CD4-APC (E-AB-F1098C, [OX-38], Elabscience Biotechnology Co., Ltd., Wuhan, China), and anti-Rat-CD3-PerCP/Cyanine (E-AB-F1098C, [G4.18], Elabscience Biotechnology Co., Ltd, Wuhan, China).

### 2.7. Blood Lipid

At the end of 10^th^ and 13^th^ weeks, the whole blood from rats in all the five groups were collected, and separately centrifuged at 2000 rpm for 20 min for serum collection. The dominant blood lipids including low density lipoprotein cholesterol (LDL-C) and total cholesterol (TCHO) were detected according to the kit instructions (LDL-C: S03029, TCHO: S03042, Rayto Co., Chemray 800, Shenzhen, China).

### 2.8. ELISA

At the end of the 13^th^ week, the whole blood from all the five groups was collected and kept at room temperature for 3-4 hours, then centrifuged at 2000 rpm for 20 min for serum collection. The sera were examined for levels of Interleukin- (IL-) 1*β* (YFXER00022, Nanjing, China), Interleukin-37 (IL-37) (INS 34412, Huangshi, Hubei, China), IL-38 (INS 30216, Huangshi, Hubei, China), N-terminal probrain natriuretic peptide (NT-proBNP) (INS 30112, Huangshi, Hubei, China), high-sensitivity cardiac troponin I (hs-cTnI) (INS 30491, Huangshi, Hubei, China), and Ischemia-modified albumin (IMA, INS 30714, Huangshi, Hubei, China).

The cardiac and cortex tissues from rats in the Control, HFD, HFD+UCMS, and HFD+UCMS+ GBE groups were collected and washed by ice-cold phosphate-buffered saline (PBS) (0.01 M, pH = 7.4) to remove the remaining blood. Then, the cardiac and cortex tissues were cut into small pieces, homogenized in PBS (1 g: 1 ml), and centrifuged to get the supernate (5 min, 5000 × g). The cardiac tissue's supernate was examined for IL-1*β* (INS 30206, Huangshi, Hubei, China), nuclear factor kappa B (NF-*κ*B) (INS 35496, Huangshi, Hubei, China), inhibitor molecule protein (I*κ*B) (INS 35702, Huangshi, Hubei, China), and IL-1 receptor (IL-1R) (INS 36885, Huangshi, Hubei, China). The cortex tissues supernate was applied for 8-iso-prostaglandinF2*α* (8-iso-PGF2*α*) (INS 15557, Huangshi, Hubei, China), which was the gold standard biomarker for excessive oxidative stress. All the ELISA detection was conducted according to the ELISA Kit instructions (YiFeiXue Biotechnology, Nanjing, China; Inselisa, Huangshi, Hubei, China).

### 2.9. Western Blot

The procedures of western blot have been described in previous research [14]. The rat's cardiac, hippocampus, hypothalamus, and cortex tissues were collected and stored at -80°C until utilized. The following three main steps for western blot technique were performed:

1. Protein extraction: the tissues were lysed with radioimmunoprecipitation assay (RIPA) lysis buffer (G2002, Servicebio, Wuhan, China) mixed with 50∗cocktail (G2006, Servicebio, Wuhan, China), phenylmethylsulfonyl fluoride (PMSF) (100 mM) (G2008, Servicebio, Wuhan, China), and phosphorylation protease inhibitor b (G2007, Servicebio, Wuhan, China), with subsequent centrifugation at 12000 rpm 4°C for 10 min. After centrifugation, the supernatants were collected and protein concentrations were measured by bicinchoninic acid (BCA) kits (G2026, Servicebio, Wuhan, China).
2. Protein separation and transfer: 10% sodium dodecyl sulfate-polyacrylamide gel electrophoresis (SDS-PAGE) was applied for separating protein; then polyvinylidene fluoride (PVDF) membranes (G6015-0.45, Servicebio, Wuhan, China) were used for transferring protein.
3. Antibody incubation and membrane analysis: the membranes were incubated with the primary antibody at 4°C overnight and then incubated with horseradish peroxidase-conjugated (HRP) secondary antibodies at room temperature for 1 hour. After incubation, the membranes were analyzed by the ELC system (6300, CLINX, Clinx Science Instruments Co. Ltd., Shanghai, China).

Primary antibodies include P65 (10745-1-ap, 1 : 1000, Wuhansanying, Wuhan, China), P-P65 (13346, 1 : 1000, Cell Signaling Technology, Boston, USA), I*κ*B*α* (ab32518,1 : 1000, Abcam, England), P-I*κ*B*α* (9246, 1 : 1000, Cell Signaling Technology, Boston, USA), glyceraldehyde-phosphate dehydrogenase (GAPDH) (GB12002, 1 : 25000, Servicebio, China), and ACTIN (GB12001, 1 : 1000, Servicebio, Wuhan, China). Secondary antibodies include: horseradish peroxidase-conjugated secondary antibodies (Servicebio GB23303, or Servicebio GB23301, Wuhan, China).

### 2.10. Oil Red Staining

Oil Red staining was carried out to evaluate the lipid deposits in the rats' aortic arteries. Prior to Oil Red staining, the aortas were fixed in 4% paraformaldehyde for more than 24 hours. The procedure was performed as follows: Firstly, after removing the fatty tissues adherent to the vascular adventitia under a stereoscope, the arteries were dissected and then pinned on a filter paper with acupuncture needles. Secondly, the samples were incubated in 60% isopropanol for 5 min, then incubated in the Oil Red stain for 60 min at 37°C. Finally, the samples were rinsed in 60% isopropanol for 5-10 min until no excess stain was seen. The lipid deposits were observed under a stereomicroscope.

### 2.11. Sirius Red Staining

Sirius Red staining was performed to display collagen fibers in the arteries. Tissue preparation involved aortic arteries fixed in 4% paraformaldehyde for more than 24 hours; then the paraffin blocks were trimmed to 3-4 *μ*m. Afterwards, to dehydrate and embed, the samples were then dehydrated with a gradient of alcohol, 70% alcohol for 1 hour, 80% alcohol for 1 hour, 95% alcohol I and II for 90 min, respectively, and 100% alcohol I and II for 1 hour, respectively; cleared in xylene I and II for 45 min, respectively; and then embedded in molten paraffin for 2 hours at 60°C. Afterwards, the slides needed preparation. The tissues were sliced to the thickness of 4 *μ*m, and the wax was melted at 60°C for 2 hours. To perform the dewaxing, the samples were immersed in xylene I and II for 10 min, respectively, then 100% alcohol I and II, 95% alcohol, and 80% alcohol for 5 min, respectively, and washed with water. Next, the samples were stained. The sections were stained with Sirius Red solution for 8 min and quickly dehydrated with 100% alcohol. Finally, the samples were dehydrated with xylene for 5 min and sealed with neutral gum. The Sirius Red stained sections were scanned by a Pannoramic scanner (Pannoramic DESK/MIDI/250/1000) and read with a Case Viewer 2.4 (3Dhistech, Hungary).

### 2.12. Hematoxylin and Eosin (HE) Staining

HE staining was applied to reveal pathological changes to arteries and cardiac tissue. Samples were fixed in 4% paraformaldehyde for more than 24 hours and followed section preparation in the same manner as Sirius Red staining, up to the point directly before staining with the Sirius Red Solution. For the hematoxylin staining, the sections were stained in the hematoxylin solution for 3-5 min, washed with running water, differentiated with differentiation solution, washed with running water again, incubated in the Hematoxylin Scott Tap Bluing and, then, finally, washed with running water. To perform the eosin staining, the sections were incubated in Eosin dye for 3 min, then washed with running water. The procedure for dehydration and sealing included placing the sections sequentially in 80% alcohol, 90% alcohol, 100% alcohol I, 100% alcohol II, xylene I and II for 5 min, respectively, then sealed with neutral gum. The HE-stained sections were then scanned by a Pannoramic scanner (Pannoramic DESK/MIDI/250/1000) and read by a Case Viewer 2.4 (3Dhistech, Hungary).

### 2.13. Immunohistochemistry

Immunohistochemical staining was employed to assess the distribution of inflammatory cytokine IL-1*β* in arteries and cardiac tissues. The specimen fixation and section preparation were the same as Sirius Red staining, up to the point directly before staining with the Sirius Red solution. Subsequently, the other steps followed were as follows: retrieving the antigen with citric acid (PH6.0) via microwave heating, blocking the activation of endogenous peroxidase, sealing with 3% bovine serum albumin (BSA), incubating with primary antibody overnight at 4°C, incubating with secondary antibody at room temperature for 50 min, 3,3′-diaminobenzidine (DAB) chromogenic reaction, counterstaining nuclei, and the previously described dehydration and sealing. The primary antibody is anti-IL-1*β* (1 : 200, Service bio, GB11113, Wuhan, China); the secondary antibody is HRP conjugated Goat Anti-Rabbit IgG (H+L) (1 : 200, Service bio, GB23303, Wuhan, China). The immunohistochemical stained sections were scanned by a Pannoramic scanner (Pannoramic DESK/MIDI/250/1000, 3DHISTECH, Hungary), read by a Case Viewer 2.4 (3DHISTECH, Hungary), and analyzed by Halo v3.0.311.314 (Indica labs, USA).

### 2.14. Transmission Electron Microscopy

Transmission Electron Microscopy (TEM) was performed to observe the ultrastructure of hippocampal cornu ammonis (CA)1 (CA1) neurons. The first step consisted of a sample preparation, where fresh hippocampus tissues were collected, and the right side of the CA1 was cut into small size pieces of 1 mm^3^ and fixed in 2.5% glutaraldehyde. The following step consisted of washing, where the samples were washed by 0.1 M PBS 3 times, 15 min each time. Subsequently, the next step consisted of a postfix, where the samples were fixed in 1% OsO_4_ for 1-1.5 hours and washed with PBS as before. Further, the next step consisted of dehydration, where a graded series of ethanol (30%, 50%, 70%, 80%, 90%, 95%, and 100%) was applied for this step, 10-15 min each time, then transferred to 100% acetone, for 10-15 min. Furthermore, the next step consisted of resin embedding, where the samples were incubated in acetone : resin (1 : 1) for 1 hour, then in acetone : resin (1 : 3) for another 3 hours, and then in pure resin for more than 5 hours or overnight. Afterwards, the samples were embedded and then polymerized at 60°C for 48 hours. Finally, the samples were cut into ultrathin sections, stained, and viewed by TEM (Tecnai G2 F20 TWIN TMP, FEI, Netherlands).

### 2.15. Statistical Analysis

All the data were presented as the mean ± standard deviation (SD) for normal distribution. Kolmogorov-Smirnov was used for analyzing data normality. One-way ANOVA was applied for three or more comparison groups, and a post hoc test LSD was used for multigroup comparisons, such as in the comparisons of serum TCHO, LDL-c, Helper T, IL-1*β*, IL-37, IL-38, hs-cTNI, NT-proBNP, IMA, LVEF, LVFS, MVE, MVA, and MVE/A among the groups of the Control, Control+UCMS, HFD, HFD+UCMS, HFD+UCMS+GBE, and in the comparisons of cardiac IL-1*β*, IL-1R, NF-*κ*B, and I*κ*B among the groups of the Control, HFD, HFD+UCMS, and HFD+UCMS+GBE. *P* < 0.05 was considered to be significant. IBM SPSS statistics software (version 24.0, IBM, Armonk, New York, USA) and GraphPad Prism Software (version 8.0.2, San Diego, CA, USA) were used for data analysis. Due to this was an exploratory study, we decided the sample size by considering scientific and qualitative levels and our former animal experiment experiences [10, 13, 14].

## 3. Results

### 3.1. Metabolism Dysfunction

#### 3.1.1. Significant Weight Loss

**Figure 1:**
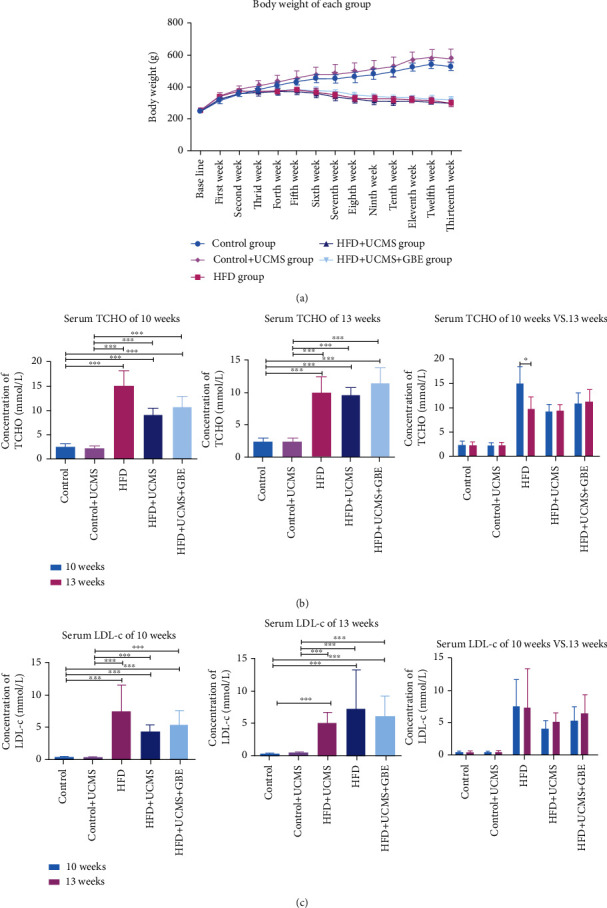
Lipid metabolism alterations. (a) The body weight of rats in different groups; (b, c) TCHO and LDL-C comparisons among the five groups in the 10th or 13th week. TCHO: total cholesterol; LDL-C: low density lipoprotein cholesterol; *n* = 5 rats per group; ^∗^*P* < 0.05;  ^∗∗^*P* < 0.01;  ^∗∗∗^*P* < 0.001.

Rat body weight was registered at the end of each week. Initially, the weight of all rats increased swiftly. However, beginning in the sixth week, the rats fed HFD began to lose weight significantly per week. In contrast, those on the normal diet continued gaining weight until the 13^th^ week (13^th^ week, Control:532.60 ± 25.77, Control+UCMS: 583.40 ± 51.90 vs. HFD: 299.80 ± 14.58, HFD+UCMS: 299.20 ± 12.03, HFD+UCMS+GBE: 315.00 ± 24.81 g, *P* < 0.05). The weight loss appeared to rule out the abnormal metabolism of rats with HFD (Figure 1(a)).

#### 3.1.2. Lipid Metabolic Alterations

By the 10^th^ week, rats with HFD had significantly higher TCHO (Control: 2.57 ± 0.61, Control+UCMS: 2.36 ± 0.47, vs. HFD: 15.19 ± 3.21, HFD+UCMS: 9.20 ± 1.44, HFD+UCMS+GBE: 10.78 ± 2.25 mmom/l, all *P* < 0.05) and higher level of LDL-c (Control: 0.42 ± 0.07, Control+UCMS: 0.40 ± 0.04, vs. HFD: 7.61 ± 4.06, HFD+UCMS: 4.30 ± 1.02, HFD+UCMS+GBE: 5.36 ± 2.18 mmom/l, all *P* < 0.05) than the control ones. By the 13^th^ week, rats with HFD had significantly higher TCHO than the controls (Control: 2.45 ± 0.47, Control+UCMS: 2.40 ± 0.48, vs. HFD: 10.01 ± 2.24, HFD+UCMS: 9.51 ± 1.13, HFD+UCMS+GBE: 11.3 ± 2.40 mmom/l, all *P* < 0.05) and LDL-c levels (Control: 0.37 ± 0.05, Control+UCMS: 0.49 ± 0.05, vs. HFD: 7.27 ± 6.16, HFD+UCMS: 5.18 ± 1.44, HFD+UCMS+GBE: 6.50 ± 2.87 mmom/l, all *P* < 0.05). Based on the results, GBE did not appear to alter changes in lipid metabolism induced by HFD (Figures 1(b) and 1(c)).

#### 3.1.3. Pathological Changes in Arteries

**Figure 2:**
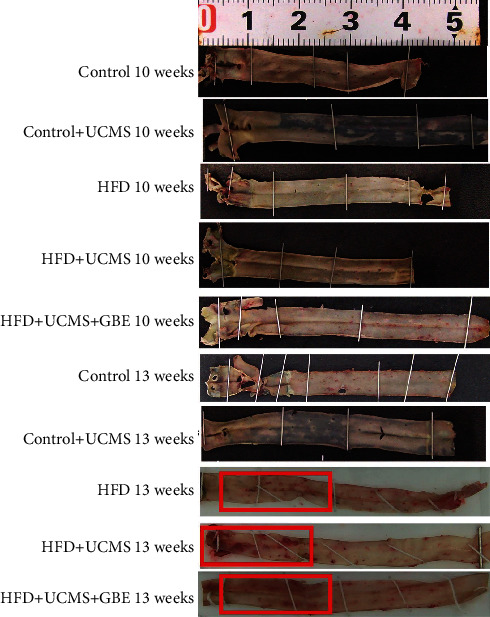
Oil Red staining of vessels. Oil Red staining of the entire aorta in different groups; the red rectangle presents lipid deposits.

**Figure 3:**
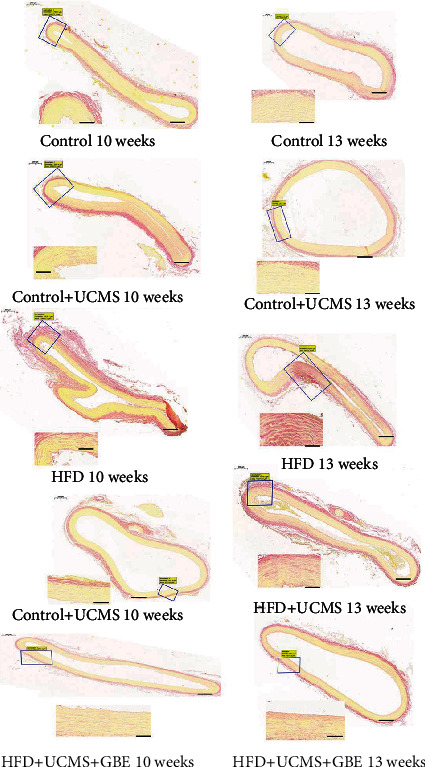
Picrosirius red staining of vessels. Picrosirius red staining of aorta ascendens sections in different groups, the red color indicates collagen fibers; the scale bar: 2000 *μ*m for the entire section and 200 *μ*m for the local section.

The Oil Red staining and Picrosirius Red staining showed that rats in HFD, HFD+UCMS, and HFD+UCMS+GBE of 13 weeks exhibited obvious lipid deposits by Oil Red staining (Figure 2) and collagen fibers by Picrosirius Red staining in the arteries (Figure 3). GBE was not shown to reduce lipid deposits and collagen fibers in rats with HFD+UCMS.

### 3.2. Protective Effect of GBE Peripheral Immune System

#### 3.2.1. Peripheral Immune Cell Determination

**Figure 4:**
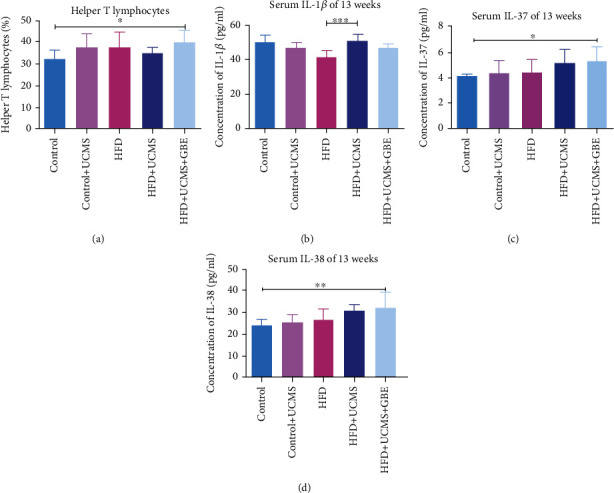
Peripheral immune imbalance. (a) The flow cytometry of TH cells for the different treatment groups at 13 weeks; (b–d) comparisons of serum inflammatory cytokines IL-1*β*, IL-37, and IL-38. Helper T lymphocytes: TH cells. *n* = 5 rats per group; ^∗^*P* < 0.05;  ^∗∗^*P* < 0.01;  ^∗∗∗^*P* < 0.001.

Flow cytometry at 13 weeks was used to detect alterations in circulating immune cells, assaying for TH cells. Compared to controls, a 13-week HFD+UCMS+GBE increased TH cells. This suggests that GBE influenced the anti-inflammatory function (Figure 4(a)). To investigate interrelationships between these immune cells and inflammatory cytokines, we consequently measured serum cytokine levels.

#### 3.2.2. Serum Proinflammatory Cytokines

In the 13^th^ week, HFD treatment alone decreased IL-1*β* (Control: 50.42 ± 4.03, Control+UCMS: 47.23 ± 2.77 vs. HFD: 42.00 ± 3.86 pg/ml, *P* < 0.05), while rats treated with HFD+UCMS for 13 weeks had higher levels of IL-1*β* than the HFD group (HFD+UCMS: 51.21 ± 3.54 vs. HFD: 42.00 ± 3.86 pg/ml, *P* < 0.05). These results indicate that UCMS could cause IL-1*β* elevation in HFD rats; however, GBE decreased the elevation of IL-1*β*, and the statistical result displayed no significance (HFD+UCMS+GBE: 47.69 ± 1.89 vs. HFD+UCMS: 51.21 ± 3.54 pg/ml, *P* > 0.05) (Figure 4(b)).

#### 3.2.3. Serum Anti-inflammatory Cytokine Levels

By the 13^th^ week, IL-37 (Control: 4.04 ± 0.19 vs. HFD+UCMS+GBE: 5.40 ± 0.96 pg/ml, *P* < 0.05) and IL-38 (Control: 25.51 ± 3.19, vs. HFD+UCMS+GBE: 34.29 ± 7.25 pg/ml, *P* < 0.05) levels were found to be increased in HFD+UCMS+GBE rats, indicating GBE cotreatment could exert effects on immune homeostasis by elevating the level of anti-inflammatory cytokines (Figures 4(c) and 4(d)).

### 3.3. Cardiac Immune Protective Effect of GBE via the Canonical NF-*κ*B Signaling Pathway

#### 3.3.1. Cardiac Dysfunction Induced by HFD, but Not Reversed by GBE

**Figure 5:**
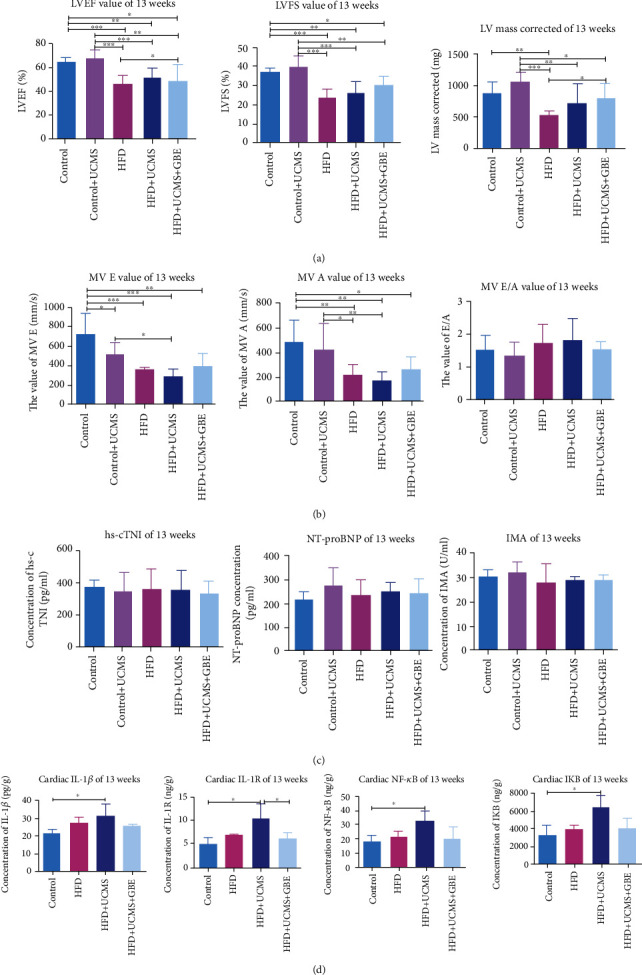
Cardiac function by echocardiography and inflammation by ELISA. (a, b) LVEF, LVFS, MVE, MVA, and MVE/A comparisons in different treatment groups in the 13^th^ week; (c) the level of hs-cTNI, NT-proBNP, and IMA comparisons in different treatment groups in the 13^th^ week; (d) comparisons of cardiac inflammatory cytokines by ELISA involving IL-1*β*, IL-1R, NF-*κ*B, and I*κ*B; IMA: Ischemia-modified albumin; LVEF: left ventricular ejection fraction; LVFS: left ventricular fractional shortening; MV E: peak E wave of Mitral valve; MV A: peak A wave of Mitral valve; *n* = 5 per group; ^∗^*P* < 0.05;  ^∗∗^*P* < 0.01;  ^∗∗∗^*P* < 0.001.

Echocardiography indicated that a HFD for 13 weeks resulted in depressed cardiac function. As compared with the control rats at 13 weeks, rats given HFD and HFD+UCMS displayed significantly lower values of LVEF (HFD: 47.18 ± 6.22%, HFD+UCMS: 50.13 ± 9.09% vs. Control: 65.60 ± 3.03%, Control+UCMS: 68.69 ± 6.07%, all *P* < 0.05), LVFS (HFD: 24.43 ± 3.86%, HFD+UCMS: 26.50 ± 5.84% vs. Control: 37.14 ± 2.52%, Control+UCMS: 40.04 ± 5.11%, all *P* < 0.05), LV mass corrected (HFD: 518.50 ± 54.52, vs. Control: 885.56 ± 148.59, Control+UCMS: 1064.00 ± 146.70 mg, all *P* < 0.05), MVE (HFD: 358.06 ± 28.73, HFD+UCMS: 290.73 ± 67.75, HFD+UCMS+GBE: 407.41 ± 114.01 vs. Control: 724.74 ± 216.17 mm/s, HFD+UCMS: 290.73 ± 67.75, vs. Control+UCMS: 511.70 ± 119.60 mm/s, all *P* < 0.05), and MVA (HFD: 217.29 ± 78.76, HFD+UCMS: 168.39 ± 63.75, HFD+UCMS+GBE: 266.57 ± 96.46, vs. Control: 490.15 ± 172.51, Control+UCMS: 421.90 ± 210.50 mm/s, all *P* < 0.05), but there was no significance in the comparisons of MV E/A (HFD: 1.79 ± 0.50, HFD+UCMS: 1.85 ± 0.60, HFD+UCMS+GBE: 1.57 ± 0.24, vs. Control: 1.54 ± 0.36, Control+UCMS: 1.36 ± 0.38, *P* > 0.05) (Figures 5(a) and 5(b), supplementary figure 1).

#### 3.3.2. No Significant Differences of Cardiac Biomarkers

At 13 weeks, there were no significant differences among the five groups of hs-cTNI (Control: 369.15 ± 48.64, Control+UCMS: 348.90 ± 113.80, HFD: 369.15 ± 112.91, HFD+UCMS: 359.30 ± 110.72, HFD+UCMS+GBE: 336.82 ± 69.78 pg/ml, *P* > 0.05), NT-proBNP (Control: 217.99 ± 26.95, Control+UCMS: 267.60 ± 77.07, HFD: 236.24 ± 63.44, HFD+UCMS: 249.29 ± 39.18, HFD+UCMS+GBE: 242.55 ± 55.59 pg/ml, *P* > 0.05), and IMA (Control: 30.65 ± 2.48, Control+UCMS: 32.02 ± 4.14, HFD: 28.03 ± 7.41, HFD+UCMS: 29.03 ± 1.13, HFD+UCMS+GBE: 29.09 ± 2.38U/ml, *P* > 0.05) (Figure 5(c)).

#### 3.3.3. GBE Attenuated Cardiac Inflammation via NF-*κ*B Signaling Pathway

The ELISA detection of immune markers in cardiac tissue indicated that, by the 13^th^ week, HFD+UCMS treatment significantly elevated the levels of IL-1*β* (31.39 ± 6.23, vs. 21.73 ± 1.94 pg/g, *P* < 0.05), IL-1R (10.90 ± 2.64, vs. 5.23 ± 1.34 ng/g, *P* < 0.05), NF-*κ*B (32.75 ± 7.22, vs. 18.51 ± 4.59 ng/g, *P* < 0.05), and I*κ*B (6360.98 ± 1246.96, vs. 3365.06 ± 1025.38 pg/g, *P* < 0.05) when compared to the control group. Meanwhile, the elevation could be reversed by GBE as evidenced (HFD+UCMS+GBE vs. HFD+UCMS, IL-1*β*: 25.87 ± 0.79 vs. 31.39 ± 6.23 pg/g, *P* > 0.05; IL-1R: 6.36 ± 1.33 vs. 10.90 ± 2.64, *P* < 0.05; NF-*κ*B: 21.17 ± 7.37 vs. 32.75 ± 7.22 pg/g, *P* > 0.05; I*κ*B: 4065.25 ± 1214.26 vs. 6360.98 ± 1246.96 pg/g, *P* > 0.05) (Figure 5(d)).

**Figure 6:**
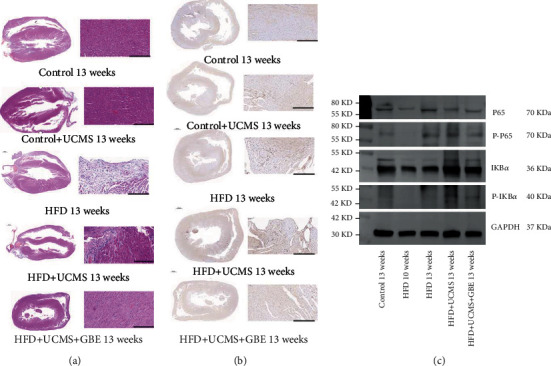
Cardiac function by pathology and inflammation by western blotting. (a) The HE-stained sections of cardiac muscles indicate there were a variety of lymphocytes in the cardiac sections of HFD+UCMS rats and HFD rats; scale bar: 10000 *μ*m and 500 *μ*m; (b) immunohistochemical staining of myocardia showing the distribution of IL-1*β*; scale bar: 10000 *μ*m and 500 *μ*m; (c) the expression of P65, P-P65, I*κ*B, and P-I*κ*B in cardiac tissue by western blot.

The HE-stained sections demonstrated that inflammatory cells, especially lymphocytes, were discovered in the myocardial muscle tissue of the rats exposed to HFD alone or with UCMS after 13 weeks. These inflammatory cells could be responsible for altered heart function, with decreased lymphocyte infiltration displayed after treatment with GBE (Figure 6(a)).

The immunohistochemical staining indicated that, after 13 weeks, the cardiac muscle of HFD rats and that of HFD+UCMS expressed a denser staining for IL-1*β* than controls, but less expression was found in HFD+UCMS+GBE animals. The positive area of IL-1*β* was quantified by Halo v3.0.311.314 (Indica labs, USA) and expressed as a percentage of the whole staining section (Control 22.87%, Control+UCMS 20%, HFD 24.71%, HFD+UCMS 46.04%, and HFD+UCMS+GBE 26.05%) (Figure 6(b)).

In accordance with the above results, western blot was performed to explore the canonical NF-*κ*B signaling pathway in cardiac tissues. The western blot showed that HFD+UCMS increased the expressions of P-P65, which could be attenuated by GBE (P-P65/GAPDH, Control: 0.21, HFD 10 weeks: 0.00, HFD 13 weeks: 0.41, HFD+UCMS: 0.46, HFD+UCMS+GBE: 0.27) and P-I*κ*B-*α* (P-I*κ*B-*α*/GAPDH, Control: 0.23, HFD10weeks: 0.01, HFD13weeks: 0.18, HFD+UCMS: 0.41, HFD+UCMS+GBE: 0.22) (Figure 6(c)).

### 3.4. Effect of GBE Treatment on Depressive Dehaviors and Brain Inflammation

#### 3.4.1. GBE Reduced Depressive Behaviors in HFD and UCMS-Treated Rats

**Figure 7:**
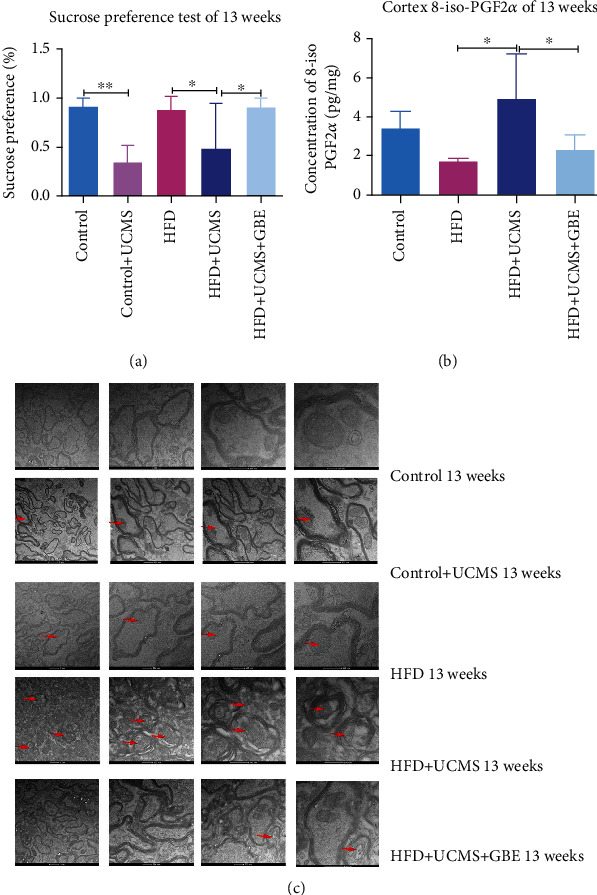
Depressive behavior and hippocampus and cortex inflammation. (a) Detection of depressive behavior by sucrose preference test; (b) the concentration of cortex 8-iso-PGF2*α*; (c) TEM demonstrating normal control tissue and treated tissue illustrating neuronal injury; the arrows show mitochondrial injuries in the hippocampus; scale 1 *μ*m and 500 nm; TEM: Transmission Electron Microscope. *n* = 5 rats per group; ^∗^*P* < 0.05;  ^∗∗^*P* < 0.01;  ^∗∗∗^*P* < 0.001.

Thirteen-week treatment with UCMS or HFD+UCMS led to depressive behaviors compared to control or HFD rats, respectively, indicated by a significantly lower preference for sucrose water. The depressive behavior could be reversed by GBE. (Control: 92.21 ± 6.93%, vs. Control+UCMS: 33.98 ± 17.19%, *P* < 0.05; HFD: 87.01 ± 14.05%, vs. HFD+UCMS: 49.12 ± 44.45%, vs. HFD+UCMS+GBE: 90.96 ± 7.51%, *P* < 0.05) (Figure 7(a)).

#### 3.4.2. GBE Relieved Cortex Oxidative Stress

In the 13^th^ week, when compared to the control group, HFD decreased the concentration of 8-iso-PGF2*α* (Control: 3.36 ± 0.85 vs. HFD: 1.69 ± 0.16 pg/mg, *P* < 0.05). When compared to the HFD group, HFD+UCMS increased the concentration of 8-iso-PGF2*α*, but was downregulated by GBE (HFD: 1.69 ± 0.16 vs. HFD+UCMS: 4.89 ± 2.36 vs. HFD+UCMS+ GBE: 2.38 ± 0.70 pg/mg, *P* < 0.05). (Figure 7(b)).

#### 3.4.3. GBE Attenuated Hippocampal Injury

Transmission Electron Microscopy (TEM) was performed to investigate the potential brain injury underlying the depressive behaviors, which were observed in Control+UCMS and HFD+UCMS rats at the end of the 13^th^ week. TEM was chosen as a reliable method to reveal the ultrastructure of hippocampal neurons. Compared to the control rats, obvious severe hippocampal CA1 neuronal injury, represented by mitochondrial swelling and disruption and autolysis in TEM images, were found in the Control+UCMS, HFD, and HFD+UCMS rats, and the severity was worsened by a combination of UCMS with HFD. However, this hippocampal nerve injury could be reversed by GBE (Figure 7(c)).

#### 3.4.4. GBE Attenuated Hippocampus, Hypothalamus, and Cortex Inflammation via the Canonical NF-*κ*B Signaling Pathway

**Figure 8:**
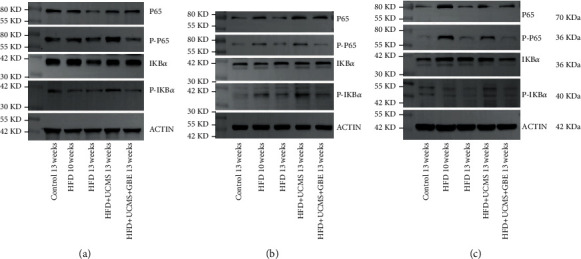
Hippocampus, cortex, and hypothalamus inflammation by western blotting. (a) The expression of P65, P-P65, I*κ*B, and P-I*κ*B in the hippocampus by western blot; (b) the expression of P65, P-P65, I*κ*B, and P-I*κ*B in the cortex by western blot; (c) the expression of P65, P-P65, I*κ*B, and P-I*κ*B in the hypothalamus by western blot.

Due to the improvement of depressive behaviors in the 13^th^ week, we conducted a western blot of the hippocampus, hypothalamus, and cortex proteins in rats with a 13-week treatment. The western blot demonstrated that the HFD+UCMS increased the expressions of P-P65 and P-I*κ*B-*α*, inducing an inflammatory response in the hippocampus (P-P65/Actin, Control: 0.57, HFD 10 weeks: 0.59, HFD 13 weeks: 0.52, HFD+UCMS: 0.97, HFD+UCMS+GBE: 0.33; P-I*κ*B-*α*/Actin, Control: 0.37, HFD 10 weeks: 0.24, HFD 13 weeks: 0.23, HFD+UCMS: 0.46, HFD+UCMS+GBE: 0.25) (Figure 8(a)), cortex (P-P65/Actin, Control: 0.13, HFD 10 weeks: 0.21, HFD 13 weeks: 0.13, HFD+UCMS: 0.30, HFD+UCMS+GBE: 0.12; P-I*κ*B-*α*/Actin, Control: 0.31, HFD 10 weeks: 0.55, HFD 13 weeks: 0.35, HFD+UCMS: 0.83, HFD+UCMS+GBE: 0.39) (Figure 8(b)), and hypothalamus (P-P65/Actin, Control: 0.11, HFD 10 weeks: 0.44, HFD 13 weeks: 0.14, HFD+UCMS: 0.27, HFD+UCMS+GBE: 0.12; P-I*κ*B-*α*/Actin, Control: 0.12, HFD 10 weeks: 0.08, HFD 13 weeks: 0.08, HFD+UCMS: 0.12, HFD+UCMS+GBE: 0.09) (Figure 8(c)). Results also demonstrate that GBE could reverse the elevation of the four proteins, implicating the role of GBE in relieving the brain inflammation via the canonical NF-*κ*B signaling pathway in the hippocampus, cortex, and hypothalamus. (Figures 8(a)–8(c)).

## 4. Discussion

Our study demonstrated that a high-fat diet supplemented by chronic stress can constitute risk factors implicated in development of cardiac and brain inflammation, both of which could be mitigated by GBE via the NF-*κ*B signaling pathway.

GBE is an extract of the tree *Ginkgo Biloba*. The main components of GBE include terpenoids, flavonoids, and organic acids. Abundant evidence has identified the therapeutic efficacy of GBE in cardiovascular and psychiatric diseases independently [15]. Therefore, we have carried out studies to address the effect of GBE on simultaneously improving the depressive behaviors of MI rats, by relieving brain inflammation via transcription 3 (STAT3) pathway [10].

We successfully established HFD+UCMS models by administering 13-weeks of HFD and UCMS. The weight loss of rats treated with a high-fat diet in this study may be attributed to the high fat fodder in their diet which included propylthiouracil. Propylthiouracil, an antithyroid drug, is used to treat Graves disease and hyperthyroidism [16]. It could promote the absorption of cholesterol and accelerate the formation of lipid plaque. As propylthiouracil also depresses thyroid function, the exposed rats develop loss of appetite and are less active, thereby leading to weight loss.

Normally, there is a strong association between immune cells and inflammatory cytokines. When triggered by inflammatory factors, immune cells start to participate in immune-related inflammation [17]. In this study, TH cells could be elevated by GBE, indicating that GBE strengthened the anti-inflammatory effect significantly.

From this study, we suggest that GBE cotreatment can provide a cardioprotective effect, though GBE could not elevate the value of LVEF in rats with HFD+UCMS. We found a significant reduction in cardiac infiltration by immune cells in HFD+UCMS+GBE animals compared with those treated with HFD and HFD+UCMS. We propose that there are several potential mechanisms: (1) We suggest that an imbalance of proinflammatory and anti-inflammatory signals contributes to myocardium disruption. GBE could suppress an inflammatory response by upregulating the expression of peripheral anti-inflammatory cytokines including IL-37 and IL-38, while downregulating the expression of proinflammatory cytokines, namely, IL-1*β* in cardiac tissues. (2) As previously demonstrated, IL-1*β* and related signaling pathways promote atherosclerosis; therefore, blocking IL-1*β* could ameliorate atherogenesis [18] and attenuate the NF-*κ*B signaling used by GBE to reduce myocardial inflammation.

Furthermore, GBE also attenuates depressive behavior (anhedonia) induced by UCMS+HFD rats. We report that the nanostructures of the right hippocampus CA1 revealed by TEM display neuronal injury, which includes mitochondrial swelling, disruption, and autolysis in HFD fed animals which was subsequently worsened by UCMS. The hippocampus, a vital structure of the limbic system [19], exhibits a close relationship with emotion, memory, and learning and is vulnerable to mental stress [20, 21], owing to its association with the HPA axis [22]. The hippocampus is divided into CA1, CA2, CA3, and dentate gyrus (DG). The CA1 has been reported to be linked with depression [23]. Accordingly, we chose to focus on the CA1 in this study. Our results are consistent with previous research demonstrating that GBE exhibited a neuroprotective effect through reduction of mitochondrial damage. In HFD+UCMS rats receiving GBE cotreatment, hippocampal neuronal injury and mitochondrial disruption are reduced (Figure 6), suggesting a potential connection between depressive behaviors and hippocampal injury which may be reduced by GBE.

GBE attenuates cortex oxidative stress induced by HFD+UCMS, through elevating of the concentration of 8-iso-PGF2*α*, which is the product of oxidative stress [24]. Oxidative stress promotes an inflammatory response, which could potentially damage the function of the brain, leading to neurodegenerative and neuropsychiatric disorders, etc. [25]. The hypothalamus secretes corticotropin-releasing hormone (CRH) and is the main component of the hypothalamus-pituitary-adrenal (HPA) axis. Therefore, the hypothalamus plays a vital role in regulating the neuroendocrine system while dealing with psychological stress [26]. Some specific regions of the cortex are implicated in the development of depression, such as the orbitofrontal cortex [27] and the anterior cingulate cortex [28]. Inflammatory factors disturb the synthesis of serotonin (5-HT) in the central nervous system, ultimately contributing to depression [29, 30].

The NF-*κ*B signaling pathway, including the canonical and noncanonical pathways, is well known in the inflammation responses [31]. In the canonical pathway, NF-*κ*B is a dimer of P65/P50. NF-*κ*B is a transcriptional factor, which combines with I*κ*B, forming a compound in the cytoplasm. Once the compound is phosphorylated, NF-*κ*B and I*κ*B separate from each other. Following this, NF-*κ*B enters the nucleus to exert the transcriptional effect. In the nucleus, NF-*κ*B activates the replication of pro-inflammatory factors in the DNA, contributing to an acute inflammation response, which can be observed by the expression of P-I*κ*B-*α* and P-P65 [31]. In addition, GBE has been reported to exert a suppressed effect on the NF-*κ*B pathway in gastric cancer mouse models [32]. In this study, the elevation of P-I*κ*B-*α* and P-P65 indicates the canonical NF-*κ*B signaling pathways of cardiac and brain tissue are activated in rats with HFD+UCMS, whereas the treatment of GBE relieves the inflammation via inhibiting the NF-*κ*B signaling pathway.

Therefore, the inflammation of the hippocampus, hypothalamus, and cortex could lead to neuroendocrine dysregulation resulting in depression. GBE could reverse the brain inflammation and attenuate the depressive behaviors via inhibiting the NF-*κ*B signaling pathway.

This study suggests there is close association between cardiac dysfunction (Figures 5 and 6) and brain injury (Figures 7 and 8) of rats fed a high-fat diet and chronic stress. Rats with lower cardiac function present depressive behaviors attribute to hippocampus injury. Similarly, ApoE-/- mice fed a high-fat diet showed atherosclerosis coexisting with depressive behaviors via hippocampus and prefrontal cortex inflammation [33]

There are some limitations in the present study. Despite GBE, we did not use anti-inflammatory or antidepressant drugs to perform comparisons, such as NF-*κ*B signaling pathway inhibitors, or Selective Serotonin Reuptake Inhibitors (SSRIs). And we only explored canonical NF-*κ*B signaling pathway in this study, without considering the noncanonical pathway. Therefore, in the next step of our experiment plan, we will conduct further study according to our current results.

## 5. Conclusions

Our study offers tantalizing hope of clinical implications: This study displays that GBE has a promising effect in treating animals in which HFD coexists with depression by reducing cardiac and brain inflammation via the canonical NF-*κ*B signaling pathway.

## Data Availability

Data and materials have been provided in the sections of methods and results.

## Abbreviations

CAD: Coronary artery disease
GBE: Ginkgo Biloba Extract
HFD: High-fat diet
UCMS: Unpredictable chronic mild stress
IL-37: Interleukin-37
LVEF: Left ventricular ejection fraction
LVFS: Left ventricular fractional shortening
MV E: Peak E wave of Mitral valve
MV A: Peak A wave of Mitral valve
LDL-C: Low-density lipoprotein cholesterol
TCHO: Total cholesterol
TH cells: Helper T lymphocytes
HE: Hematoxylin and Eosin
TEM: Transmission Electron Microscope
SD: Standard deviation
DG: Dentate gyrus.

## Supplementary Materials

Supplementary Materials Supplementary figure 1: cardiac function by echocardiography. (A) The figures presenting LVEF and LVFS. (B) The figures presenting MV E and MV A. LVEF: left ventricular ejection fraction; LVFS: left ventricular fractional shortening; MV E: peak E wave of Mitral valve; MV A: peak A wave of Mitral valve.
