# Supplementary figures and images for: Ginkgo Biloba Extract Reduces Cardiac and Brain Inflammation in Rats Fed a HFD and Exposed to Chronic Mental Stress through NF-κB Inhibition
Source: Mediators Inflamm. 2022 May 29;2022:2408598. doi: 10.1155/2022/2408598 (PMC9168192; doi:10.1155/2022/2408598)

Supplementary figure 1

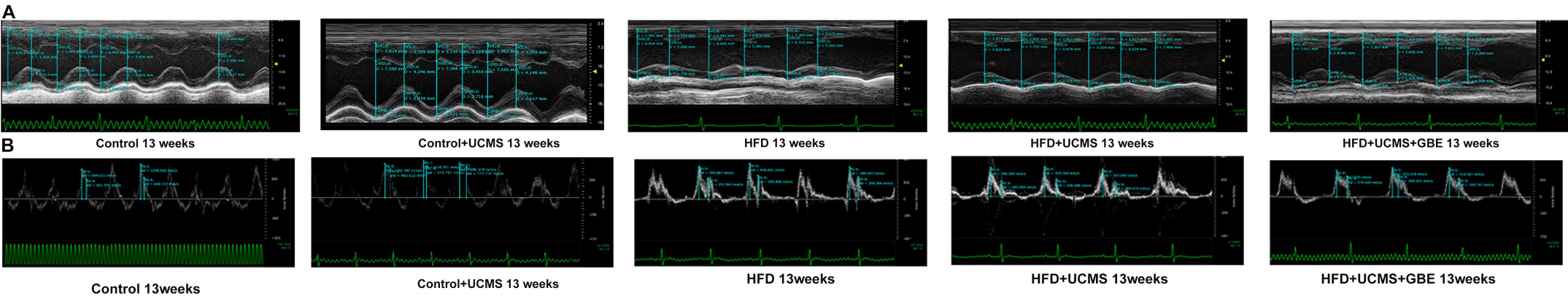

Supplement: Supplementary Materials — Supplementary figure 1: cardiac function by echocardiography. (A) The figures presenting LVEF and LVFS. (B) The figures presenting MV E and MV A. LVEF: left ventricular ejection fraction; LVFS: left ventricular fractional shortening; MV E: peak E wave of Mitral valve; MV A: peak A wave of Mitral valve. [file 2408598.f1.pdf]
